# Lumbar Intervertebral Disc and Discovertebral Segment. Part 2: An Imaging Review of Pathologic Conditions With Anatomic Correlation

**DOI:** 10.7759/cureus.25733

**Published:** 2022-06-07

**Authors:** Daphne J Theodorou, Stavroula J Theodorou, Ioannis D Gelalis, Yousuke Kakitsubata

**Affiliations:** 1 Radiology, MRI-CT Unit, General Hospital of Ioannina, Greece, Ioannina, GRC; 2 Radiology, Musculoskeletal MRI, University Hospital of Ioannina, Ioannina, GRC; 3 Orthopedics, University Hospital of Ioannina, Ioannina, GRC; 4 Radiology, Musculoskeletal Imaging, Miyazaki Konan Hospital, Miyazaki, JPN

**Keywords:** cadavers, lumbar spine, disc disease, abnormalities, discovertebral segment, intervertebral disc

## Abstract

The lumbar intervertebral disc is a complex anatomic structure that can be affected by a number of distinct pathologic processes. Categories of the disease include degenerative changes, subclinical or overt trauma, infectious lesions, inflammatory insults, metabolic disease, and tumors. Abnormalities affecting the intervertebral disc may assume atypical appearances or alterations may as well mimic pathologic processes related to degeneration that can be asymptomatic. Although the imaging findings of degenerative diseases of the vertebral column have been emphasized extensively, the assembly of pathologic conditions associated with the discovertebral segment has not received adequate attention. This manuscript reviews and illustrates a range of abnormalities affecting the discovertebral segment, providing a detailed analysis of postmortem material, in the realm of a close anatomic-imaging correlation. Knowledge of the characteristic morphology and patterns of abnormal conditions affecting the intervertebral disc and discovertebral segment can help radiologists narrow the differential diagnosis in a broad spectrum of disease processes.

## Introduction

Low back pain is regarded as the most disabling condition in the world, in addition, is associated with high medical expenditures and significant psychosocial consequences. Lumbar pain increases in prevalence with age; it reaches a lifetime prevalence of 85% and causes disability in up to 8% of the adult population. MR imaging has become the imaging tool of choice that allows direct visualization of major anatomic structures in the musculoskeletal system, offering detailed anatomic information that complements physical examination. In previous studies, MR imaging has proven that can depict principal abnormalities affecting the discovertebral segment in exquisite detail, similar to that disclosed in anatomic studies of cadavers [1].

Although degenerative processes occurring in the lumbar spine have been invariably associated with low back pain the clinical relevance of disc degeneration at MR imaging in patients with low back pain remains to be determined [2]. Unfortunately, a range of distinct disc abnormalities, including disc degeneration, disc herniation, and annular tears have been shown to be common in asymptomatic volunteers and as such, the ultimate significance ascribed to MR imaging has been questioned [3,4].

The primary use of imaging in patients with lumbar pain is to identify the location of the chief complaint in any or all of the spinal articulations and supporting structures, and direct appropriate management. Internal derangements of the lumbar disc (e.g., fissuring or tearing of the annulus fibrosus), changes in the facet joints, spinal ligaments, and paravertebral soft tissues are included among the most common degenerative related pathologic processes that are associated with spinal pain and disability [5]. In addition, spinal cord compression (myelopathy) and nerve root compression (radiculopathy) generate pain related to osteophytosis, altered spinal mechanics due to intervertebral disc displacement, and degenerative spondylolisthesis and segmental instability, or a combination of confounding factors [6]. The spectrum of pathologic conditions intrinsic to the spine that may affect the intervertebral disc and discovertebral segment includes an extended group of discrete degenerative processes, trauma (acute or chronic), ischemia, infectious and inflammatory conditions, and neoplasm (e.g., metastasis). These abnormalities conclude in a range of histologic and imaging patterns of discovertebral disease that have not been fully addressed in the medical literature and may provide meaningful clues to the pathogenesis of significant discogenic pain. To explore the various patterns of discovertebral aberrations, we undertook a correlative imaging-pathologic study using data from autopsy specimens.

## Materials and methods

**Figure 1:**
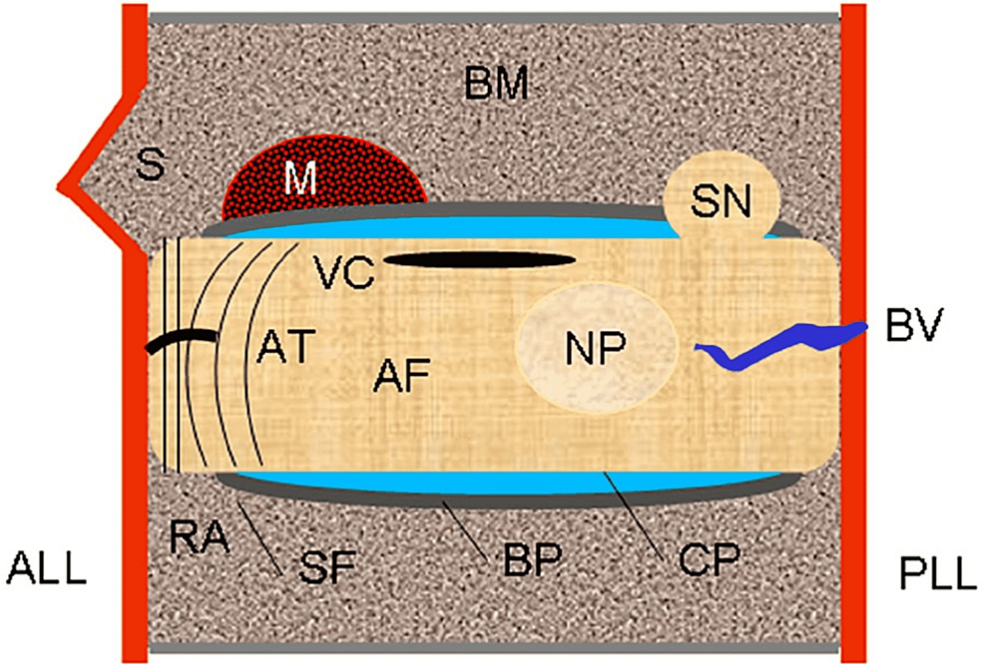
Discovertebral segment. Schematic drawing shows configuration of disc and sites of major abnormalities. NP = nucleus pulposus, AF = annulus fibrosus, CP = cartilaginous plate, BP = bone plate, RA = ring apophysis, SF = Sharpey fibers, AT = annular tear, VC = vacuum cleft, SN = Schmorl node, M = metastasis, S = spur, BM = bone marrow, ALL = anterior longitudinal ligament, PLL = posterior longitudinal ligament, BV = basivertebral vein (drawing by S.J.T).

The anatomy of the spine is depicted in several excellent atlases; however, its correlation to several derangements has not been widely reviewed. Data derived from anatomic studies in cadavers, however, are recognized as the foundation of research because they provide major clues to the documentation of the pathogenesis of clinically significant abnormalities [1,5]. Coupled with MR imaging findings, the diverse disease findings affecting the spinal structures are noteworthy [1]. In this manuscript, we illustrate the various patterns of abnormalities we have encountered involving the lumbar discovertebral segments (n= 320) of 65 dissected human cadavers to provide a practical framework for the clinical recognition of disease processes that can lead to low back pain. Understanding the diverse and distinct anatomic abnormalities underlying discovertebral disease helps focus on the true source of symptoms emphasizing discogenic pain, and can narrow the entertained broad, differential diagnostic considerations of lumbar pain (Figure 1).

## Results

Discovertebral abnormalities: imaging - pathologic correlation

**Figure 2:**
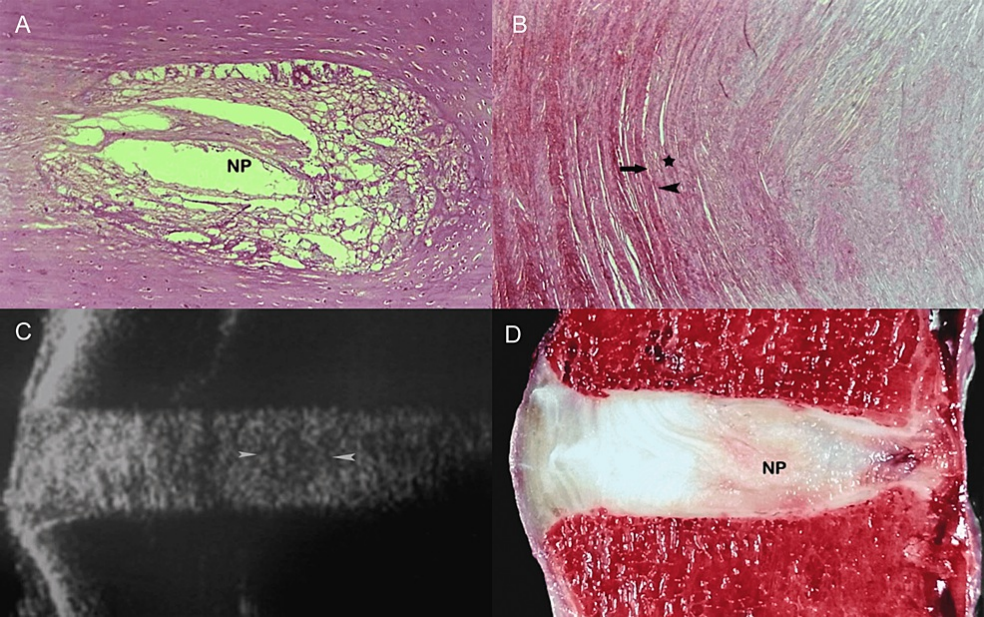
A 29-week-old premature fetus lumbar spine. (A) Sagittal anatomic section proves that the boundary between the nucleus pulposus (NP) and annulus fibrosus is distinct. There are notochordal remnants in the nucleus which contain abundant amorphous mucoid material and are surrounded by fibrocartilage of the annulus fibrosus. (B) Histologic section of the annulus fibrosus. Observe the pattern of transition of the fibers at the interface (asterisk) between the multilayered outer fibers (arrowhead) and the inner annular fibers (arrow) (H&E, objective x20). (C) Sagittal sonographic image delineates echogenic structure of the intervertebral disc. The nucleus pulposus appears relatively hypoechogenic (arrowheads) and it is differentiated from the remainder of the disc. (D) Corresponding anatomic section in the sagittal plane shows well demarcated NP surrounded by the annulus fibrosus.

Like elsewhere in the spine, the lumbar intervertebral disc consists of an inner nucleus pulposus, surrounded by the peripheral annulus fibrosus [1,5]. Normal discs, in young persons, are composed mainly of water which comprises almost 90% of the nucleus pulposus and 80% of the annulus fibrosus. Proteoglycans and collagen form an extracellular matrix whose function is twofold: first, to attract water molecules and generate osmotic pressure that expands the disc, and second, to maintain structural support allowing the annulus to resist the radial tension imposed by axial loading. In young individuals, the nucleus pulposus has a gelatinous consistency and is easily discernible from the annulus fibrosus [1,5]. The annulus fibrosus consists of peripheral, concentric laminae of collagenous fibers and an inner zone of fibrocartilage (Figures 2A-2D).

**Figure 3:**
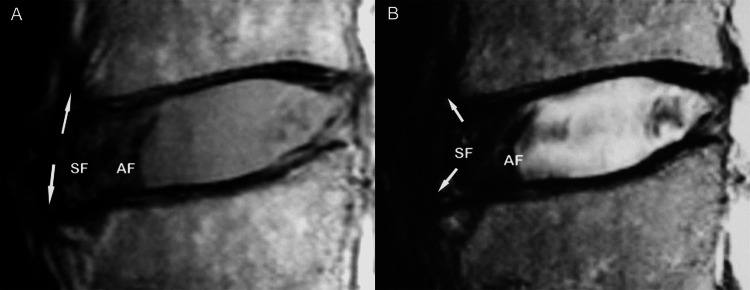
Sharpey fibers on MRI. Sagittal T1-weighted (A) and T2-weighted (B) MR images display the site of origin (thin arrows) of strong Sharpey fibers (SF) at the periphery of the annulus fibrosus (AF), in the region of the ring apophysis.  This is the exact site of anchorage of the annulus to the anterior vertebral surface.

The annulus is attached to the cartilaginous end-plate and the vertebral rim by calcified cartilage, while it is anchored to the periosteum of the apposing vertebral bodies via strong Sharpey fibers (Figures 3A, 3B).

**Figure 4:**
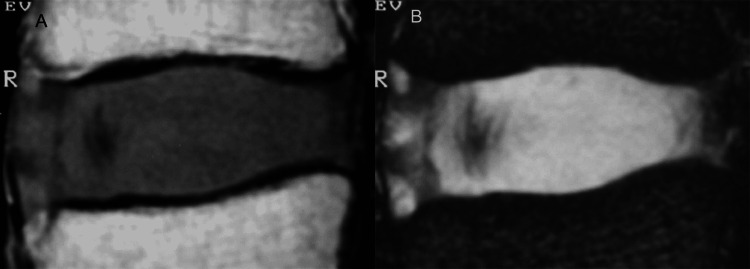
The adult disc. (A) Sagittal T1-weighted MR image in a 43-year-old cadaveric specimen shows the intervertebral disc with intermediate signal intensity. The nucleus pulposus is not visible. (B) Corresponding sagittal T2-weighted MR image shows the intervertebral disc with predominant high signal intensity. The nucleus pulposus cannot be distinguished from the remainder of the disc.

 With aging, the nucleus becomes stiffer and is difficult to be differentiated from the degenerating annulus (Figures 4A, 4B) [1].

**Figure 5:**
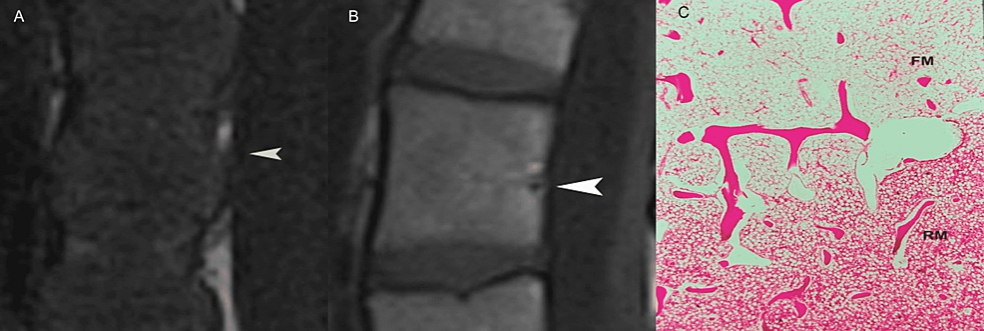
Vertebral bone marrow. (A) Sagittal T1-weighted MR image in a newborn exhibits the diffuse decreased signal intensity of the vertebral marrow. Differentiation of marrow from the disc is poor. Arrowhead points to the basilar vein. (B) Sagittal T1-weighted MR image in adults shows T1 high signal intensity within cancellous bone of the lumbar vertebra.  Differentiation of marrow from the disc is clear. Note vascular channels (arrowhead). (C) Histologic section discloses physiologic transition between red (RM) and fatty marrow (FM) during conversion, in a different specimen (not shown).

In each discovertebral segment, physiologic age-related changes also occur in the intimately related vertebral marrow, with red marrow be gradually replaced by yellow marrow. In healthy subjects, progressive conversion of red to yellow marrow is reflected on MR images with increasing signal intensity within the vertebral bodies (Figures 5A-5C).

**Figure 6:**
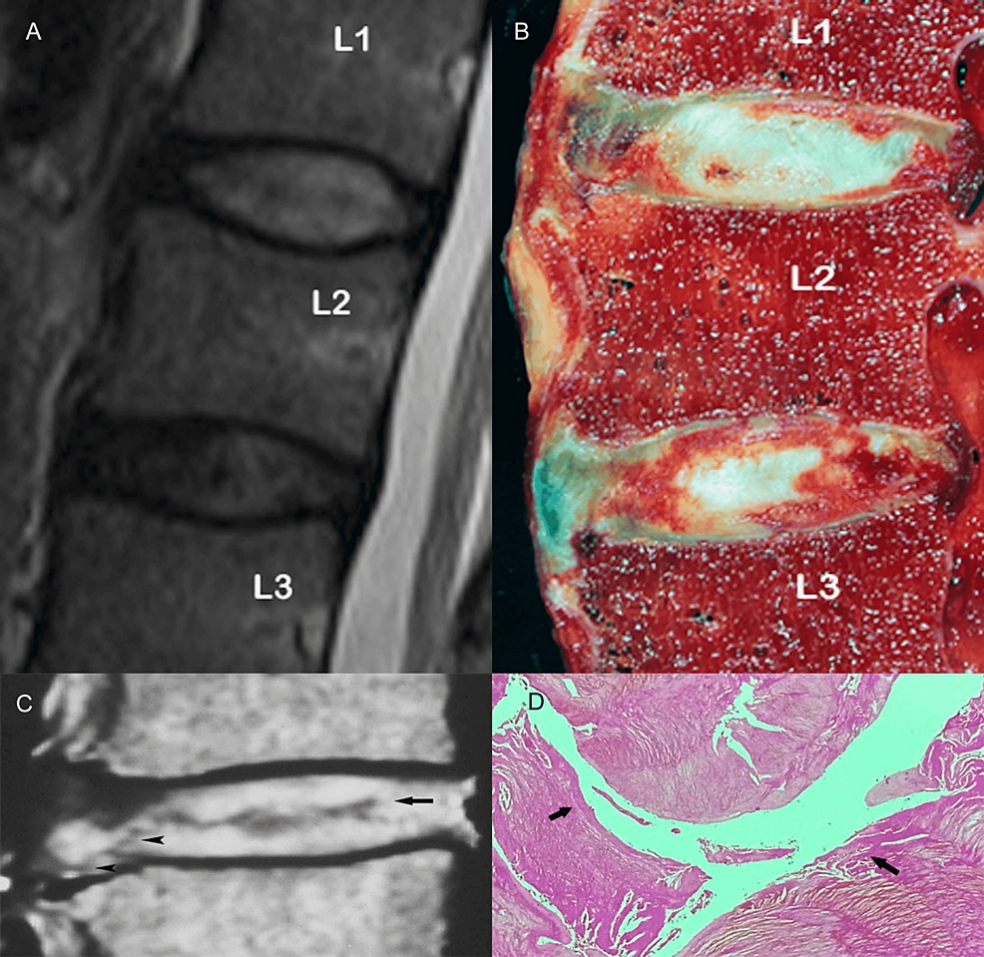
The intranuclear cleft. (A) Sagittal T2-weighted MR image shows the intervertebral disc at the L1-L2 interspace of high signal intensity relative to that of the disc at the L2-L3 level. (B) Corresponding anatomic section depicts a normal disc at the L1-L2 level, whereas the disc at the L2-L3 appears grossly abnormal. (C) Sagittal T2-weighted MR image (another case) shows a large, transverse wavy area (arrow) of low signal intensity consistent with a nuclear cleft.  Note that the crevice within the disc substance extends more peripherally involving first, the inner fibers and later, the outer fibers of the annulus fibrosus from the interior to the exterior of the disc (arrowheads). (D) Corresponding (to figure part C) histologic section reveals frank cleft (arrows) branching in the disc.

Among the earliest imaging findings of degenerative disc disease are disc dehydration and diminution of disc height [1,7]. The normal intervertebral disc appears on T1-weighted MR images as an elliptical structure of intermediate signal intensity and relatively high signal intensity on T2-weighted images. With aging decreasing proteoglycans within the disc attract less water molecules, which coupled with changes in the structure and synthesis of collagen impair dramatically the biochemical and biomechanical properties of the disc [8]. These alterations of the biochemical content in turn, result in disc desiccation seen as hypointensity on T2-weighted images and loss of the disc height [5,7]. Diminished osmotic pressure affects the mechanical competence of the disc to withstand compressive loads and may be associated with the formation of intranuclear clefts observed on T2-weighted images as zones of decreased signal intensity when compared to the nucleus pulposus (Figures 6A-6D) [1].

**Figure 7:**
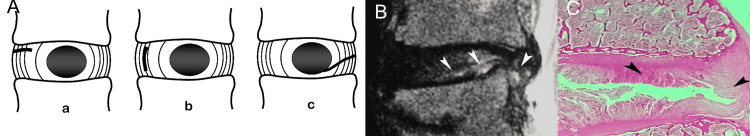
Annular tears. (A) Schematic drawing shows the classification of annular tears into three major types. a = peripheral tear (rim lesion), representing eccentric horizontal tear of the outer annular fibers; b = circumferential tear (concentric lesion), representing concentric splitting of the annular lamellae; and, c = radial tear, representing horizontal tear that extends from the nucleus pulposus to the outer portion of the annulus fibrosus. Drawing by Y.K. (B) Sagittal T2-weighted MR image shows annular tears as linear areas of high signal intensity within the disc, extending to the posteriorly herniated disc material (arrowheads). (C) Corresponding histologic section displays discal tear (arrowheads).

In the continuum of biochemical and morphological changes occurring with disc desiccation, impaired tissue resiliency affects functional mechanics of the disc and predisposes the annulus to form fissures or tears [9]. Annular tears are categorized as peripheral (rim lesions), circumferential (concentric lesions), and radial and may be seen on T2-weighted images as high-intensity zones coursing within the annular substance (Figures 7A-7C) [2].

**Figure 8:**
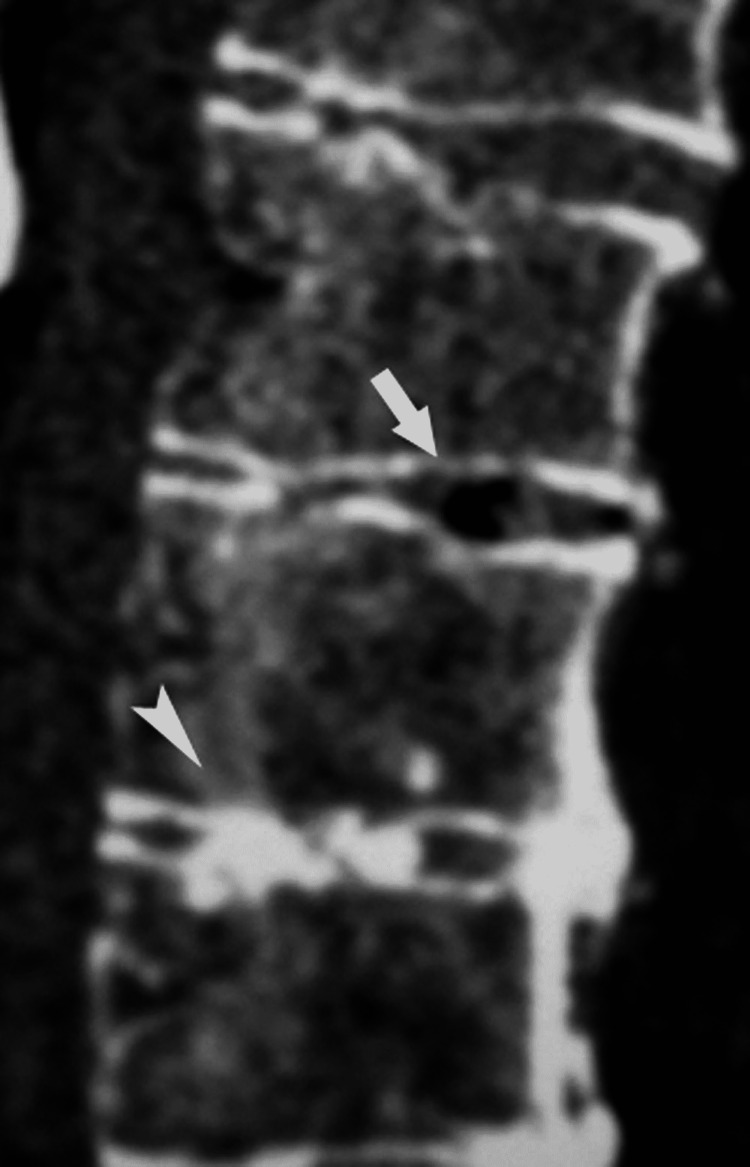
Vacuum disc. A reformatted sagittal CT image reveals a radiolucent area consistent with a vacuum phenomenon in the disc (arrow). Note the calcification of the upper disc (arrowhead).

As the disc degenerates gas typically nitrogen, accumulates in fissures or clefts that form within the nucleus and extend into the annulus fibrosus. Within these abnormal defects, negative pressure is generated, producing “vacuum” disc phenomena [1,5]. Intradiscal gas collection (vacuum disc) appears as a signal void on MR images (Figure 8) [1,10,11].

**Figure 9:**
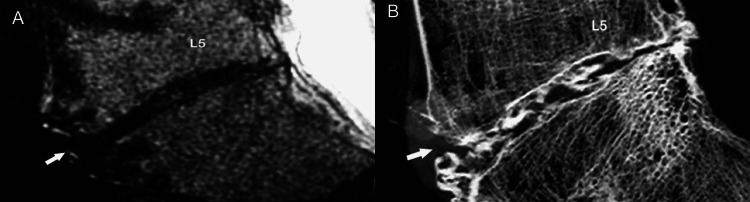
Disc calcification. (A) Sagittal T2-weighted MR image shows predominant low signal intensity of the intervertebral disc (arrow) at the markedly narrowed L5-S1 interspace. (B) Corresponding low-kilovoltage contact radiograph reveals densely calcified disc (arrow) at that level. There is loss of disc height, eburnation of adjacent bone, and spur formation.

Degenerative disc calcification is another common manifestation of degenerative disc disease. Calcified areas are usually amorphous or can assume a curvilinear configuration adjusted to the shape and orientation of the annular fibers [12]. In most cases, calcifications are displayed as low signal intensity on both the T1- and T2-weighted images (Figures 8, 9A, 9B).

**Figure 10:**
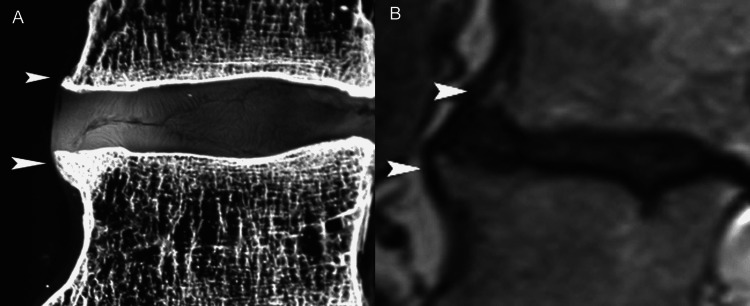
Osteophytosis. (A) Sagittal slab contact radiograph shows prominent osteophytes (arrowheads) in the margin of the vertebral bodies. (B) Sagittal MR image reveals thick cortex and fat marrow in the spurs (arrowheads).

Osteophytes are osseous excrescences that form due to altered biomechanics at the site of anchorage of the annulus fibrosus to the vertebral body, in the discovertebral junction [13,14]. These bony outgrowths, found in at least 60% to 80% of the elderly, may be asymptomatic or may be associated with mechanical pain due to affected kinematics that essentially curtail spinal function (Figures 10A, 10B).

**Figure 11:**
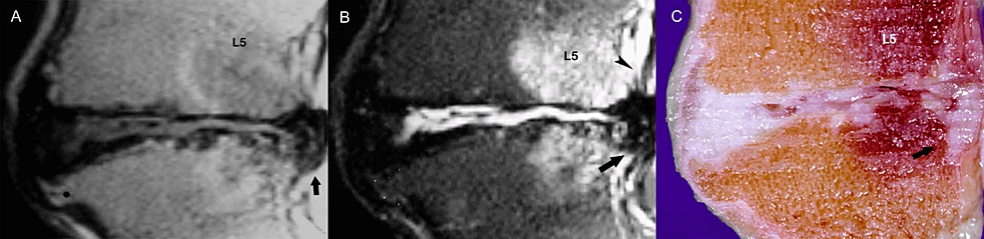
Disc herniation. (A) Sagittal T1-weighted MR image shows severe disc degeneration at the L5-S1 vertebral space with posterior disc herniation (arrow). There is markedly narrowed intervertebral disc space and grossly abnormal configuration of the vertebral end-plates. Note the prominent osteophyte at S1 (asterisk). (B) Sagittal T2-weighted MR image demonstrates extruded disc material (arrow) violating the posterior longitudinal ligament (arrowhead). (C) The findings of intervertebral (osteo)chondrosis are confirmed on the corresponding anatomic section (arrow).

Advancing age is associated with many other complications of these degenerative processes. Gradual dehydration and loss of disc elasticity tends to alter resistance of the offended intervertebral disc to the exercised biomechanical overload, which as the disc deteriorates, impacts the global mechanics of lumbar spine. Loss of hydrostatic pressure weakens the annulus and urges the abnormally compressed nucleus to prolapse from its strictly confined space [8,15]. Disc displacement or herniation can generally be classified as central, lateral, or posterolateral, depending on the direction of extension of the disc material beyond the interspace, with or without compression of the spinal cord and the nerve roots [16-18]. Biomechanical overload and disc herniation occur more commonly at the lower lumbar spine (Figures 11A-11C).

**Figure 12:**
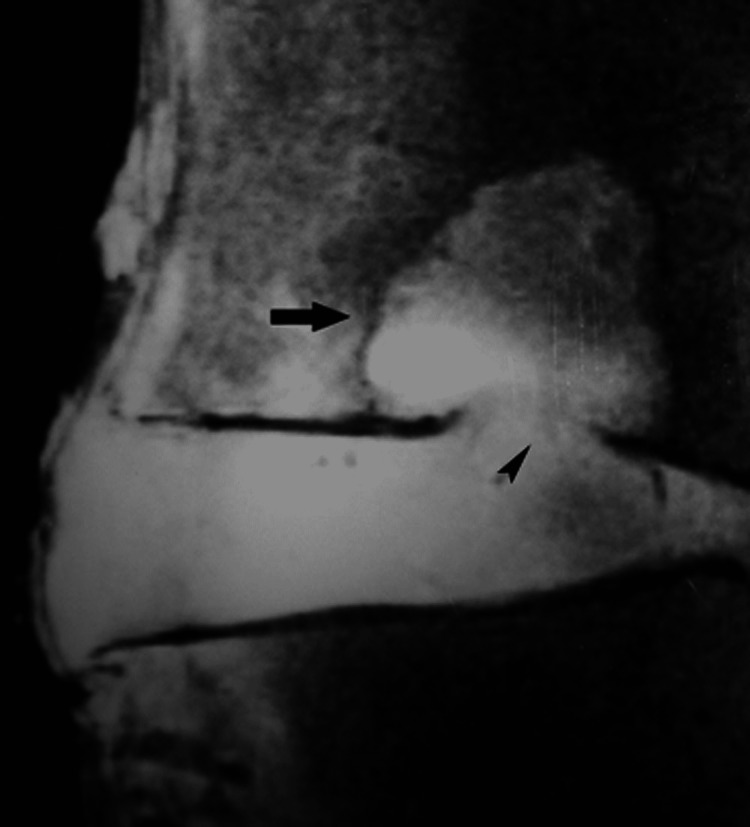
Schmorl nodes. Sagittal MR image demonstrates large cartilaginous (Schmorl) node (arrow). Note disruption of the midportion of the vertebral end-plate (arrowhead).

Under abnormal stress, progressive displacement of the nucleus pulposus may cause the nucleus to penetrate the bony end-plate, forming a cartilaginous or Schmorl node that may in turn, generate pain (Figure 12) [7].

**Figure 13:**
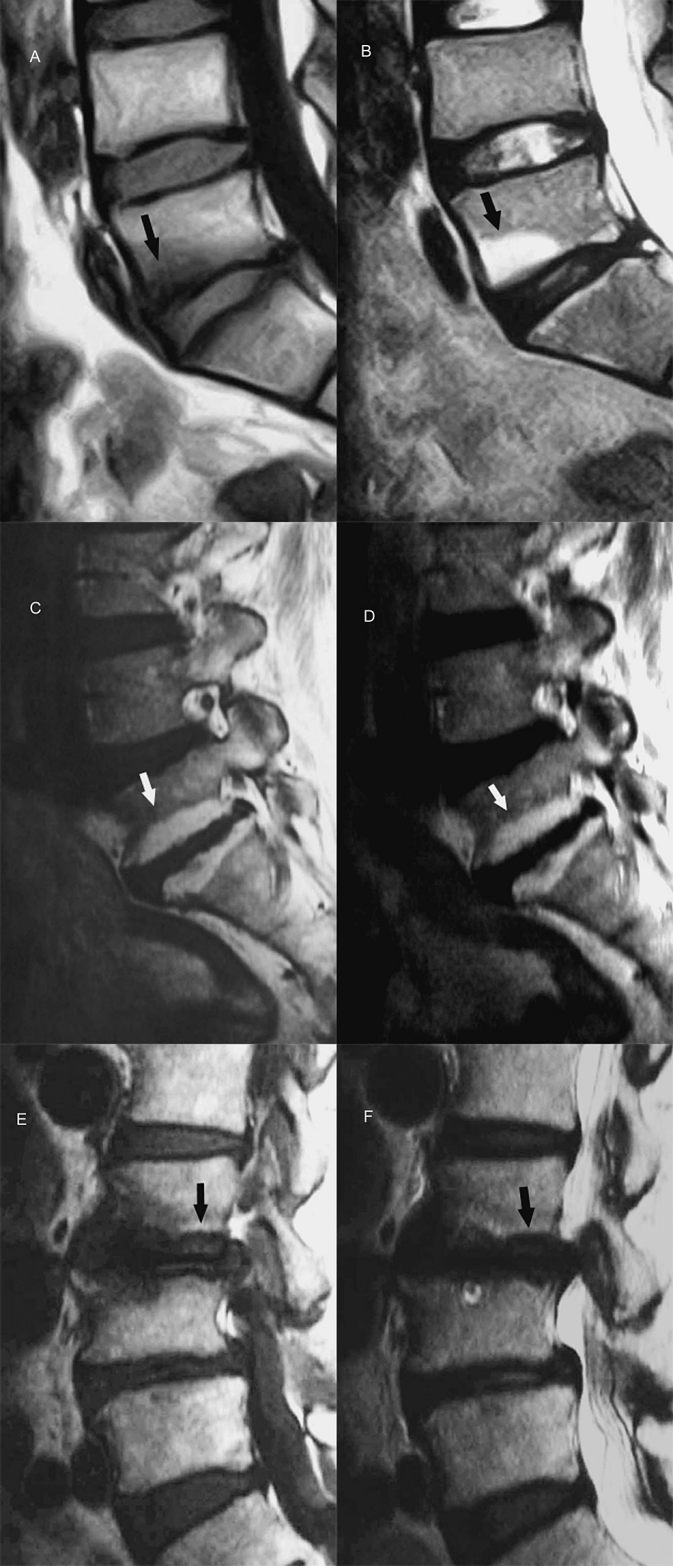
End-plate changes. (A, B) Sagittal T1- and T2-weighted MR images show Modic type 1 end-plate marrow changes (arrow) of low and high signal intensity, respectively corresponding to the formation of fibrovascular tissue. (C, D) Sagittal T1- and T2-weighted MR images show Modic type 2 end-plate marrow changes (arrow) of high signal intensity corresponding to fatty marrow. (E, F) Sagittal T1- and T2-weighted MR images show Modic type 3 end-plate marrow changes (arrow) of low signal intensity corresponding to the formation of fibro-osseous tissue.

Within the range of disc degeneration concomitant degenerative alterations in the vertebral end-plates are classified into three categories based on distinctive MR imaging characteristic [7,19-22]. Type I end-plate changes appear as low signal intensity on T1-weighted images and high signal intensity on T2-weighted images, reflecting the presence of fibrovascular tissue in the abnormal end-plate. Type II degenerative end-plate changes are visualized as high signal intensity on T1-weighted images and decreased signal intensity on T2-weighted images, owing to the deposition of adipose tissue in bone marrow. Type III end-plate changes are seen as low signal intensity on both T1-weighted and T2-weighted images, consistent with prominent hyperostosis that has replaced normal marrow (Figures 13A-13F).

**Figure 14:**
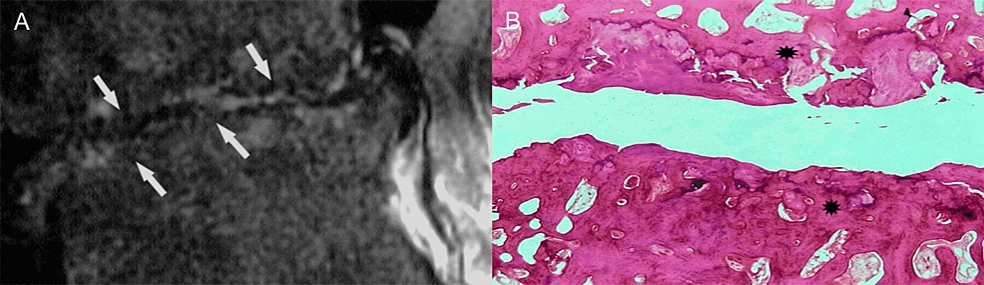
Advanced degenerative changes. (A) Sagittal T2-weighted MR image shows marked destruction of the vertebral end-plates (arrows). Predominantly irregular marrow-disc interface and remarkable loss of disc space are evident. (B) Histologic section shows prominent osteosclerotic changes of the abnormal,  degenerated vertebral end-plates (asteriks).

Because abnormalities may vary immensely in severity, advanced degeneration may conclude in significant loss or near-complete obliteration of the vertebral interspace, and gross destruction of architecture at the discovertebral segment (Figures 14A, 14B) [7].

**Figure 15:**
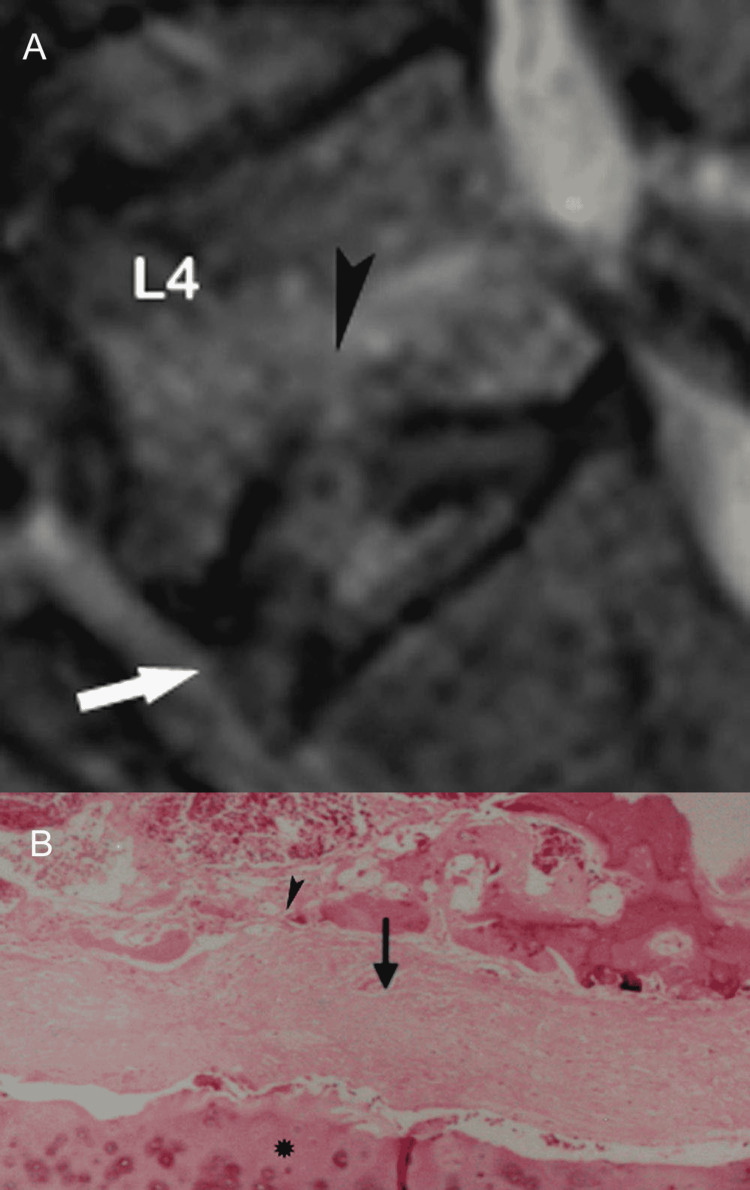
End-plate fracture. (A) Sagittal T2-weighted MR image shows destruction of the lower end-plate of fourth lumbar vertebra (arrowhead) with abnormal high signal intensity in the lower part of vertebral body and intervening disc (arrow). Upper end-plate of fifth lumbar vertebral body appears unremarkable. (B) Histology section reveals severe destruction of the bony and cartilaginous end-plate (arrowhead) at the site of fracture and adjacent disc fibrosis (arrow). Of note, the neighboring cartilaginous end-plate of fifth lumbar vertebral body is spared (asterisk).

Progressive deterioration of the end-plates, cell senescence and microvascular insufficiency, especially when associated with osteoporosis, microtrauma or macrotrauma can result in marked weakening of the bony end-plate and fracture. Eventually, the vertebral body collapses [1,7]. With MR imaging, fracture is readily depicted as end-plate discontinuity with or without collapse (Figures 15A, 15B).

**Figure 16:**
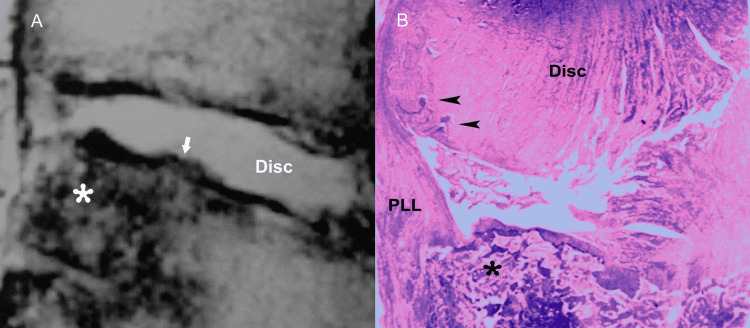
Tumorous invasion of the vertebral end-plate and disc. (A) Sagittal T2-weighted MR image shows metastatic breast tumor (asterisk) at the posterior portion of the S1 vertebral body infiltrating the vertebral endplate (arrow). (B) Histologic section reveals mixed pattern of osteolysis and osteosclerosis in trabecular bone (asterisk) at the site of tumorous deposit. Note newly formed blood vessels (arrowheads) in the infiltrated disc. PLL, posterior longitudinal ligament.

Besides traumatic disruption of the vertebral end-plate, the differential diagnosis of hyperintensity on T2-weighted images of the disc and the adjacent vertebral bodies includes infection (e.g., spondylodiscitis), inflammatory diseases (e.g., rheumatoid arthritis), and neoplasm (primary tumor or metastasis) (Figures 16A, 16B) [23-30].

The clinical context and pattern of involvement at the level of visualization of abnormal signal intensity changes generally allow for diagnosis and distinction among several clinical differential diagnostic possibilities that may cause lumbar pain.

## Discussion

In short, a plethora of disease processes can become the source of symptoms and signs in the lumbar spine, although degenerative alterations predominate. We have found distinct pathologic aberrations that are evident at the microscopic level, gross inspection of the spine as well as on the imaging studies. Basic knowledge of the imaging features of the spinal disease as presented in this manuscript is fundamental to a better understanding of the source of discogenic pain. Increased awareness of the abnormalities affecting this region will conclude in improved diagnosis and more efficient management of the processes affecting this seemingly simple, yet complex cartilaginous articulation of the spine. We recognize some weaknesses in this study on cadavers. First, we used standard MR imaging sequences to delineate abnormalities; and, conceivably we did not engage spectroscopy, diffusion-weighted imaging, or other functional MR imaging techniques that are aimed at biochemical tissue analysis and functional assessment. In our imaging protocols, we used technical parameters dedicated to a limited portion of the body, and such protocols proved useful for to direct assessment of these structures, however. Second, many specimens were harvested from the cadavers of elderly humans; therefore, some of the investigated structures may have been subjected to degeneration. This study contains the largest number of cadavers studied with MR imaging-anatomic correlation. Finally, this study is unique not only in a large number of cadavers and discovertebral segments analyzed but also in the utilization of high-resolution slab radiography, a technique that increases detailed visualization of sectional anatomy around the intervertebral disc.

## Conclusions

Dedicated high-resolution MR imaging is well suited for investigating internal derangements of the discovertebral segment and may enable confident diagnosis in most instances. In the appropriate clinical context, a wide range of abnormalities affecting the discovertebral segment can be accurately diagnosed. Radiologic evaluation of discovertebral disease may enable clinicians select treatment options for conservative or operative treatment and avoid complications.
